# Supplementary figures and images for: Modulating Cell–Scaffold Interaction via dECM-Decorated Melt Electrowriting PCL Scaffolds
Source: Polymers (Basel). 2025 Nov 25;17(23):3133. doi: 10.3390/polym17233133 (PMC12694446; doi:10.3390/polym17233133)

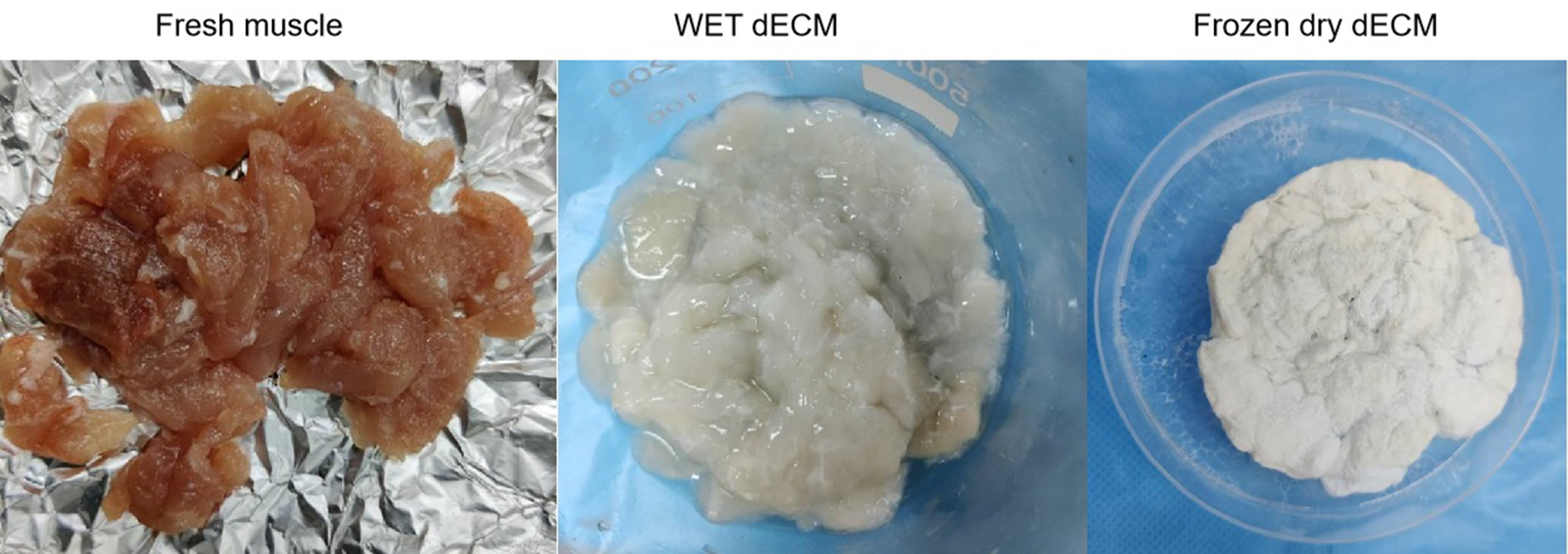

Supplement: Supplementary file 1 [file polymers-17-03133-s001.zip › polymers-3978794-supplementary.jpg]
